# Variations in outcomes for women admitted to hospital in early versus active labour: an observational study

**DOI:** 10.1186/s12884-020-03149-7

**Published:** 2020-08-17

**Authors:** Yvette D. Miller, Ashleigh A. Armanasco, Laura McCosker, Rachel Thompson

**Affiliations:** 1 Queensland University of Technology, Institute of Health & Biomedical Innovation, School of Public Health & Social Work, Kelvin Grove, Brisbane, QLD 4059 Australia; 2 School of Public Health, Faculty of Medicine and Health, The University of Sydney, Camperdown, NSW 2006 Australia

**Keywords:** Medical intervention, Birth, Hospital admission, Labour management, Active phase, Latent phase

## Abstract

**Background:**

There is no available evidence for the prevalence of early labour admission to hospital or its association with rates of intervention and clinical outcomes in Australia. The objectives of this study were to: estimate the prevalence of early labour admission in one hospital in Australia; compare rates of clinical intervention, length of hospital stay and clinical outcomes for women admitted in early (< 4 cm cervical dilatation) or active (≥4 cm) labour; and determine the impact of recent recommendations to define early labour as < 5 cm on the findings.

**Methods:**

We conducted a retrospective cohort study using medical record data from a random sample of 1223 women from live singleton births recorded between July 2013 and December 2015. Analyses included women who had spontaneous onset of labour at ≥37 weeks gestation whilst not a hospital inpatient, who had not scheduled a caesarean section before labour onset or delivered prior to hospital admission. Associations between timing of hospital admission in labour and clinical intervention, outcomes and hospital stay were assessed using logistic regression.

**Results:**

Between 32.4% (< 4 cm) and 52.9% (< 5 cm) of eligible women (*N* = 697) were admitted to hospital in early labour. After adjustment for potential confounders, women admitted in early labour (< 4 cm) were more likely to have their labour augmented by oxytocin (AOR = 3.57, 95% CI 2.39–5.34), an epidural (AOR = 2.27, 95% CI 1.51–3.41), a caesarean birth (AOR = 3.50, 95% CI 2.10–5.83), more vaginal examinations (AOR = 1.73, 95% CI = 1.53–1.95), and their baby admitted to special care nursery (AOR = 1.54, 95% CI = 1.01–2.35). Defining early labour as < 5 cm cervical dilatation produced additional significant associations with artificial rupture of membranes (AOR = 1.41, 95% CI = 1.02–1.95), assisted vaginal birth (AOR = 1.96, 95% CI = 1.12–3.41) neonatal resuscitation (AOR = 1.73, 95% CI = 1.01–2.99) and longer maternal hospital stay (AOR = 1.21, 95% CI = 1.04–1.40).

**Conclusions:**

Findings provide preliminary evidence that a notable proportion of labouring women are admitted in early labour and are more likely to experience several medical procedures, neonatal resuscitation and admission to special care nursery, and longer hospital stay.

## Background

Evidence from large international retrospective cohort studies [1–6] suggests between 10.2% [1] and 58.5% [2] of women are admitted to hospital in latent phase (‘early’) labour. International guidelines recommend a delay in hospital admission during the early phase of labour by supporting women to stay at home [7, 8]. Despite these recommendations, and the overwhelming prevalence of early labour admissions, there are prevailing gaps in research to date. While some evidence exists to suggest admission to hospital in early labour results in poorer outcomes for women and the healthcare system, there is minimal evidence for the prevalence of early labour admission in diverse settings and its association with rates of intervention and clinical outcomes is inconsistent.

Existing evidence from Canada, the USA, Italy, Finland, Iraq, and Australia suggest a relationship between premature hospital admission and obstetric intervention^.^ Women admitted to hospital in early labour are more likely to experience augmentation of labour with oxytocin [3, 4, 6, 9–12], epidural analgesia [1, 3, 6, 12], delivery by emergency or unplanned caesarean section [1–4, 9, 11–15], and instrumental or assisted vaginal delivery [3, 9, 16]. However, differences in absolute rates of these interventions between women admitted in early and active labour vary widely between studies and evidence for the relationship between the timing of hospital admission and clinical outcomes is inconsistent or inconclusive. Also, there is a paucity of Australian evidence for both the prevalence and outcomes of premature hospital admission in labour; indeed, the only Australian study [12] found reported on the relationship between timing of hospital admission in labour and just *one* outcome, caesarean section.

Adding to these inconsistencies, there is little consensus in definitions of onset of any identified stages of labour in the research literature and considerable variation in the definition applied to distinguish early from active labour [17]. Studies have defined active labour as cervical dilatation of > 3 cm [1, 2, 6, 11, 16], ≥4 cm [3–5, 13], ≥4.5cms [10] and ≥ 5cms [15] at hospital admission, or used rates of cervical dilatation during the first 4 h after admission [12]. At the time of data collection for this study, the World Health Organisation’s (WHO) ‘Diagnosis of Labour’ protocol recommended active labour defined as ≥4 cm cervical dilatation at time of hospital admission [18], but more recently recommended a shift to diagnosing active labour from 5 cm cervical dilatation [19]. At the same time, the WHO [19] affirmed lack of studies specifically investigating birth outcomes based on the use of different definitions of phases of the first stage of labour.

The mechanisms for the relationship between early hospital admission and intervention are undoubtedly complex. Davey et al. suggest temporal factors may play a role in the administration of interventions; “Once they arrive at hospital, women themselves may become impatient, and caregivers are compelled to use resources (e.g. birth suite beds) efficiently, so may be reluctant to ‘await events’.” [15] Whatever the cause, the length of hospital stay and ‘cascade’ of obstetric intervention associated with admission to hospital in early labour results in a significant cost-burden on the healthcare system; one Australian study estimated that this could increase the relative cost of birth by up to 50% for low-risk primiparous women [20].

This research addressed the gaps identified here by gathering local evidence on a comprehensive list of interventions and outcomes in women at all levels of obstetric risk. As part of a broader initiative to better inform local policy makers, clinicians, and women, the objective of this study was to establish evidence on the prevalence of admission to hospital in early labour in a single, public funded tertiary hospital and to compare maternal and neonatal clinical procedures and outcomes, and length of hospital stay, for women admitted to hospital in early and active labour. Further, we sought to determine how recent changes in WHO recommendations for defining early vs active labour affected the prevalence of admission to hospital in ‘early’ labour and associations with maternal and neonatal procedures and outcomes.

## Methods

### Study design and setting

We conducted a retrospective cohort study using data obtained from paper medical records. The study setting was Caboolture Hospital, a publicly funded tertiary hospital in a regional area of Queensland, Australia, providing maternity care to approximately 2000 women per year through both obstetric-led and midwifery-led care models. Caboolture hospital had higher proportions of women receiving midwifery-led care (43%) than publicly funded birth facilities in Queensland (35%) and other public facilities with the same Clinical Services Capability (32%) in 2015 [21]. Eligible for inclusion in the study were women who gave birth in Caboolture Hospital in three chosen time periods^1^ where women [1] had a singleton pregnancy, [2] planned to have a vaginal birth (i.e. did not have a caesarean section scheduled in advance of labour onset), [3] had spontaneous onset of labour while *not* a hospital inpatient (women admitted for birth after rupture of membranes were regarded as being in early labour at time of admission if cervical dilatation was recorded as < 4 cm), [4] were at ≥37 weeks’ gestation, [5] did not give birth prior to hospital admission [6] had a live birth and [7] had at least one vaginal examination recorded during the episode of hospital admission. The data collection periods were selected to allow secondary usage of the data for a different project utilising a time series design for evaluating upcoming service changes.

### Measures

#### Independent variable

The independent variable was stage of labour at admission. Women were compared according to whether they were in early labour (< 4 cm cervical dilatation) or in active labour (≥4 cm cervical dilatation [7]) as assessed by vaginal examination closest to time of admission. These definitions were consistent with the World Health Organisation ‘Diagnosis of Labour’ protocol and clinical practice in the study setting (and elsewhere) at the time the study was conducted [18]. To establish how recent changes in recommendations for diagnosing active labour [19] impacted on the prevalence and outcomes of admission in early labour, we also compared women using alternative definitions of early labour as < 5 cm cervical dilatation and active labour as ≥5 cm cervical dilatation.

#### Outcomes

We consulted with local clinicians to ensure the outcomes being assessed were complete and relevant to practice, within the scope of available data. We assessed the following clinical interventions and outcomes: augmentation by oxytocin, artificial rupture of membranes, epidural analgesia, other pharmacological pain relief (i.e. nitrous oxide, narcotics), use of non-pharmacological pain relief (i.e. sterile water injections, water immersion, heat packs, massage), number of vaginal examinations, episiotomy, length of maternal and infant hospital stay, caesarean birth, caesarean birth for slow progress, caesarean birth for fetal distress, assisted vaginal birth, postpartum haemorrhage (blood loss > 500 mL), neonatal resuscitation and admission of infant to special care nursery. For all relevant interventions and outcomes, local diagnoses and definitions were adopted.

#### Potential confounders

Non-modifiable maternal characteristics that might reasonably be associated with both the independent variable (stage of labour at admission) and outcomes of interest were also assessed. These characteristics comprised parity, gravidity, previous caesarean section, maternal age, and distance from residence to hospital (calculated from residential suburb and postcode to hospital address).

### Sample

We estimated the number of cases required to detect differences between women admitted in early versus active labour – in the prevalence of three key outcomes of interest: [1] caesarean section, [2] use of oxytocin to augment spontaneous labour, and [3] artificial rupture of membranes. Without knowing the actual distribution of timing of admission in the population, we assumed equal (50%) distribution of the sample across groups – that is, a 1:1 ratio of early and non-early labour admissions. We used existing rates of the three outcomes from all births with spontaneous labour onset in Caboolture Hospital in 2013 (advised by the Perinatal Data Collection, Health Statistics Unit, 17th March 2014: 16% Caesarean section, 18% oxytocic augmentation, and 30% artificial rupture of membranes).

A sampling goal of 720 eligible cases was established to allow detection of a 8% difference in caesarean section prevalence (20% vs 12%; 326 required per group) and use of oxytocin for augmentation (22% vs. 14%; 359 required per group), and a 10% difference in artificial rupture of membranes (35% vs. 25%; 326 required per group), with 80% power and 95% confidence. Caboolture Hospital records an average of 525 births per quarter, with an estimated 340 births per quarter (59%) in women who meet the eligibility criteria for this study. Therefore, the sampling goal was inflated by 41% to account for ineligibility in the selected sample and to provide sufficient statistical power (i.e. final N of 720) to detect the changes between groups in primary outcomes of interest. This resulted in a required minimum of 1220 cases.

The hospital identified all live births at ≥37 weeks’ gestation (and excluding multiple births for the 2015 period) across the sampling period (*N* = 3031). Using a random number generator, we selected a random sample of 1223 woman.

### Data analysis

First, we used separate logistic regression analyses to assess the equivalence of the groups on potentially confounding variables (i.e., parity, gravidity, previous caesarean section, maternal age, distance). Variables found to be non-equivalent between groups were considered as potential confounders. Second, we conducted a series of logistic regressions to compare the groups on clinical interventions and outcomes, adjusting for potential confounders.

We adjusted for parity in all analyses. Additional logistic regression analyses were conducted for selected outcomes – specifically, use of epidural, use of pharmacological pain relief, use of non-pharmacological pain relief, episiotomy, caesarean section for slow progress, and length of hospital stay – after additional adjustment for other outcomes that could be related to both the independent and dependent variable. For example, we included additional adjustment for augmentation with oxytocin in assessing associations between timing of labour admission and use of epidural and other pain relief, given the potential for increased pain (and subsequent use of pain relief) for the mother of the uterotonic-induced contractions associated with hyperstimulation of the uterus with oxytocic augmentation. We also included additional adjustment for augmentation with oxytocin in assessing associations with caesarean section for ‘slow progress’ given their common indication of “failure of labour to progress” [22] We additionally adjusted for mode of birth for outcomes that co-occur with type of birth (i.e., episiotomy, and length of both maternal and neonatal hospital stay; see Tables 2 and 3).

### Quality control

We used several methods to ensure the quality of data retrieved from paper medical records (similar to [23]), including a standardised data retrieval form and protocol, initial and ongoing training for all five data retrievers (three who were health care professionals with research training and two who were researchers without clinical healthcare experience), and inter-rater reliability analysis. A random selection of 10% of eligible charts (*N* = 73) were separately entered by two data retrievers. An initial visual inspection of inter-rater reliability was conducted when 14 charts had been double entered, to check for consistency in data retrieval. At least one second entry for each retriever was included. Initial checks included a visual inspection of discrepancies and percent agreement between retrievers. This data was used to update the Data Entry Manual and training for all data retrievers.

The final analysis of inter-rater reliability was conducted on a selection of key variables. Agreement between data collectors for categorical variables was assessed using Cohen’s kappa. Two-way mixed intraclass correlation coefficient (ICC) analyses were used to assess agreement among raters for continuous variables [24]. Kappa values were interpreted using recommendations provided by Shrout [25] where values of 0.81 and above are considered substantial agreement, 0.61 to 0.80 moderate, 0.41 to 0.61 fair, 0.11 to 0.40 slight and values below 0.10 virtually no agreement.

## Results

### Sample

**Fig. 1:**
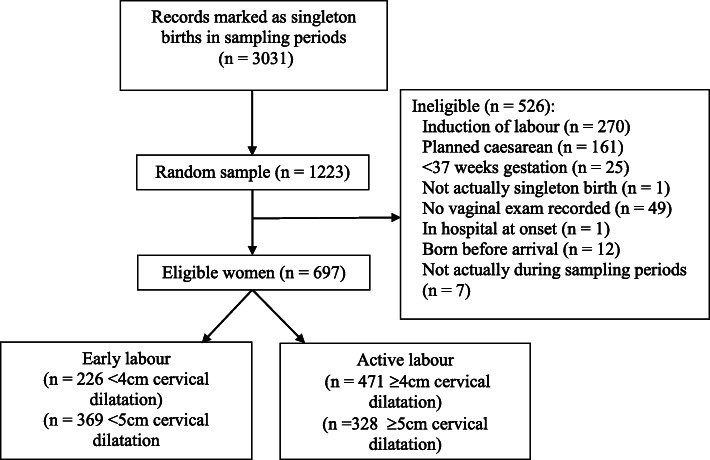
Participant flowchart

From the randomly selected sample (*N* = 1223), there were 697 (57%) eligible women. (Fig. 1). Among eligible women, 226 (32.4%) were admitted to hospital in early labour using the criteria recommended at the time of this study (< 4 cm cervical dilatation), with the remainder admitted to hospital in active labour. Parity was the only non-modifiable participant characteristic associated with stage of labour at admission (see Table 1). Women admitted in early labour were more likely to be primiparous (54.0%) than those admitted in active labour (38.5%; OR = 2.61, 95%CI [1.88, 3.62], *p* < .001).

**Table 1:** Non-modifiable participant characteristics by stage of labour at admission (*N* = 697)

Outcome	Total	Active labour (≥4 cm) (*n* = 471)	Early labour (< 4 cm) (*N* = 226)	OR^a^	95% CI	*p*
	% (n)	% (n)	% (n)			
Primiparous	38.5 (268)	31.0 (146)	54.0 (122)	2.61	1.88, 3.62	*< .001*
Primigravida	63.4 (441)	63.0 (296)	64.2 (145)	1.05	0.76, 1.46	.762
Previous caesarean	8.0 (56)	7.2 (34)	9.7 (22)	1.39	0.79, 2.43	.254
	*M (SD)*	*M (SD)*	*M (SD)*	OR^a^	95% CI	*p*
Maternal age (years)	27.94 (8.99)	28.24 (9.05)	27.34 (8.86)	0.99	0.97, 1.01	.224
Distance to hospital (km)	14.36 (14.75)	13.74 (12.74)	15.64 (18.19)	1.01	1.00, 1.02	.119

Referent category is admission in active labour

Applying the criteria of at least 5 cm of cervical dilatation at time of admission increased the proportion of women estimated to be in early labour at time of admission to 52.9%. Among women who were categorised as being admitted in active labour using the earlier criteria of > = 4 cm (*N* = 471), 30.4% were re-categorised as being in early labour using the updated recommendations of <=5 cm cervical dilatation at time of admission.

### Outcomes

After adjusting for parity, women admitted in early labour (< 4 cm cervical dilatation) were significantly more likely than those admitted in active labour to have had labour augmented by oxytocin, to have had an epidural, to have used pharmacological pain relief, to have had a caesarean birth, to have had a caesarean for slow progress, and to have had a caesarean for fetal distress (see Table 2). They also had more vaginal examinations and a longer hospital stay. Babies born to women admitted in early labour were more likely than those born to women admitted in active labour to have been admitted to the special care nursery. There were no significant associations between stage of labour at admission and artificial rupture of membranes, non-pharmacological pain relief, episiotomy, assisted vaginal birth, postpartum haemorrhage, neonatal resuscitation, or infant length of hospital stay. Additionally, some associations were no longer significant once we added adjustment for additional variables that could reasonably be associated with timing of admission and outcomes. The association between stage of labour at admission and use of pharmacological pain relief was no longer significant after adjusting for augmentation by oxytocin (see Table 2). The association between stage of labour at admission and maternal length of hospital stay was no longer significant after adjustment for mode of birth.

**Table 2:** Frequencies and adjusted odds ratios for outcomes by stage of labour at admission

Outcome	Active labour	Early labour	Odds of outcome if admitted in early labour (< 4 cm), adjusting for parity			Odds of outcome if admitted in early labour adjusted for parity and additional factors		
	% (n)	% (n)	AOR^a^	95% CI	*p*	AOR^a^	95% CI	*p*
Augmentation by oxytocin	12.8 (60)	39.8 (90)	3.57	2.39, 5.34	*< .001*			
Artificial rupture of membranes	33.0 (155)	32.4 (73)	0.95	0.67, 1.35	.776			
Epidural	15.7 (74)	40.7 (92)	3.10	2.13, 4.51	*< .001*	2.27^b^	1.51, 3.41	*<.001*
Pharmacological pain relief	70.1 (330)	83.2 (188)	1.79	1.19, 2.70	*.006*	1.52^b^	1.00, 2.32	.053
Non-pharmacological pain relief	45.4 (214)	46.5 (105)	0.89	0.64, 1.24	.495	0.90^b^	0.64, 1.27	.541
Episiotomy	8.9 (42)	15.5 (35)	1.29	0.78, 2.14	.323	1.36^c^	0.75, 2.46	.315
Caesarean birth	5.9 (28)	22.1 (50)	3.50	2.10, 5.83	*<.001*			
Caesarean for slow progress	3.6 (17)	12.8 (29)	3.03	1.60, 5.74	*.001*	2.40^b^	1.22–4.72	.011
Caesarean for fetal distress	2.3 (11)	8.4 (19)	2.91	1.33, 6.35	*.007*			
Assisted vaginal birth	8.1 (38)	15.0 (34)	1.49	0.89, 2.49	.128			
Postpartum haemorrhage	16.7 (78)	14.2 (32)	0.85	0.54, 1.33	.469			
Neonatal resuscitation	7.6 (36)	13.3 (30)	1.69	0.99, 2.86	.053			
Admission to special care nursery	13.8 (65)	21.7 (49)	1.54	1.01, 2.35	*.046*			
	*M (SD)*	*M (SD)*	AOR^a^	95% CI	*p*	AOR^a^	95% CI	*p*
Number of vaginal examinations	2.20 (1.40)	3.69 (1.75)	1.73	1.53, 1.95	*<.001*			
Maternal length of stay (days)	1.95 (1.29)	2.52 (1.30)	1.25	1.09, 1.42	*.001*	1.13^d^	0.98, 1.30	.102
Neonate’s length of stay (days)	1.78 (1.31)	2.10 (1.31)	1.08	0.95, 1.22	.233	0.97 ^d^	0.85, 1.12	.690

^a^Referent category is admission in active labour

^b^Adjusted for parity and augmentation with oxytocin

^c^Adjusted for parity and mode of birth (vaginal, assisted vaginal or caesarean)

^d^Adjusted for parity and mode of birth (vaginal or caesarean)

Defining early labour as cervical dilatation < 5 cm produced similar associations between stage of labour at admission and outcomes reported for early labour defined as < 4 cm cervical dilatation above (see Table 3). However, stage of labour at admission was significantly associated with artificial rupture of membranes, assisted vaginal birth, and neonatal resuscitation (after adjustment for parity), and there was no longer a significant association between stage of labour at admission and caesarean for fetal distress. Further, the significant association with use of pharmacological pain relief was sustained after additional adjustment for augmentation by oxytocin, and the association with maternal length of stay remained after additional adjustment for mode of birth (Table 3), where these associations disappeared with additional adjustment in the models applying definitions of early labour as cervical dilatation < 4 cm.

**Table 3:** Frequencies and adjusted odds ratios for outcomes by stage of labour at admission where early labour is defined as < 5 cm cervical dilatation

Outcome	Active labour	Early labour	Odds of outcome if admitted in early labour (< 5 cm), adjusting for parity			Odds of outcome if admitted in early labour adjusted for parity and additional factors		
	% (n)	% (n)	AOR^a^	95% CI	*p*	AOR^a^	95% CI	*p*
Augmentation by oxytocin	9.2 (30)	32.8 (120)	3.99	2.54, 6.28	*< .001*			
Artificial rupture of membranes	28.8 (94)	36.4 (134)	1.41	1.02, 1.95	.040			
Epidural	9.1 (30)	36.9 (136)	5.05	3.26, 7.82	*< .001*	3.91^b^	2.47, 6.18	*<.001*
Pharmacological pain relief	64.6 (212)	82.9 (306)	2.34	1.63, 3.35	*< .001*	2.01^b^	1.39, 2.91	*<.001*
Non-pharmacological pain relief	45.1 (148)	46.3 (171)	0.92	0.68, 1.25	.600	0.91^b^	0.67, 1.26	.582
Episiotomy	7.9 (26)	13.8 (51)	1.34	0.79, 2.25	.278	1.30^c^	0.77, 2.21	.331
Caesarean birth	5.2 (17)	16.5 (61)	2.84	1.60, 5.05	*<.001*			
Caesarean for slow progress	2.4 (8)	10.3 (38)	3.63	1.65, 7.99	*.001*	2.64^b^	1.16–5.98	.020
Caesarean for fetal distress	2.1 (7)	6.2 (23)	2.34	0.97, 5.61	*.058*			
Assisted vaginal birth	6.1 (20)	14.1 (52)	1.96	1.12, 3.41	.018			
Postpartum haemorrhage	14.8 (48)	16.8 (62)	1.21	0.79, 1.83	.383			
Neonatal resuscitation	6.7 (22)	11.9 (44)	1.73	1.01, 2.99	*.048*			
Admission to special care nursery	12.2 (40)	20.1 (74)	1.63	1.07, 2. 05	*.024*			
	*M (SD)*	*M (SD)*	AOR^a^	95% CI	*p*	AOR^a^	95% CI	*p*
Number of vaginal examinations	1.87 (1.14)	3.41 (1.74)	2.16	1.86, 2.50	*<.001*			
Maternal length of stay (days)	1.85 (1.24)	2.40 (1.33)	1.30	1.13, 1.49	*.001*	1.21^d^	1.04, 1.40	.011
Neonate’s length of stay (days)	1.72 (1.26)	2.03 (1.35)	1.11	0.98, 1.25	.110	1.03 ^d^	0.91, 1.18	.610

^a^Referent category is admission in active labour

^b^Adjusted for parity and augmentation with oxytocin

^c^Adjusted for parity and mode of birth (vaginal, assisted vaginal or caesarean)

^d^Adjusted for parity and mode of birth (vaginal or caesarean)

There were insufficient cases to assess maternal or neonatal transfer to a higher care facility or maternal or neonatal death.

### Inter-rater reliability

Inter-rater reliability was assessed in a random sample of 72 records of eligible women. Kappa statistics for categorical variables ranged from 0.81 to 0.95 (Table 4), indicating substantial agreement (κ ≥ 0.81). Continuous variables had substantial agreement (ICC ≥ 0.81; number of vaginal examinations and infant length of hospital stay) to perfect agreement (maternal length of hospital stay) between data collectors (Table 4).

**Table 4:** Inter-rater reliability for categorical and continuous variables

Variable	n	κ	
Stage of labour at admission	72	0.88	
Augmentation with oxytocin	70	0.81	
Artificial rupture of membranes	71	0.86	
Epidural	72	0.90	
Episiotomy	71	0.89	
Mode of birth	72	0.93	
Postpartum haemorrhage	72	0.92	
Neonatal resuscitation	70	0.82	
Admission to SCN	70	0.95	
	n	ICC	95% CI
Number of vaginal examinations	71	0.91	0.86, 0.94
Maternal hospital stay in days	67	1.00	1.00, 1.00
Infant hospital stay in days	64	0.99	0.99, 0.99

## Discussion

Nearly a third (32.4%) of women who had a live, singleton birth at ≥37 weeks gestation with spontaneous onset of labour and without a caesarean section scheduled in advance of labour onset, were admitted in early labour (< 4 cm cervical dilatation). Applying the criteria of at least 5 cm of cervical dilatation at time of admission increased the proportion of women admitted in early labour to 52.9%. Primiparous women were more likely to be admitted in early labour than multiparous women. After adjusting for parity, hospital admission during early labour was significantly associated with several clinical interventions (oxytocic augmentation, artificial rupture of membranes, epidural, pharmacological pain relief, caesarean birth and caesarean for slow progress, assisted vaginal birth, vaginal examinations, neonatal resuscitation), neonatal admission to special care and maternal length of hospital stay.

In this first Australian assessment of the prevalence of hospital admission in early labour, the identified rate of 32.4% falls at approximately the midpoint of the 10.2 to 58.5% range found in previous retrospective cohort studies in Sweden [1], Iran [2], Canada [3], the United States [4], Bangladesh [5] and Italy [6]. Our estimates of the 52.9% prevalence of hospital admission in early labour when applying the criteria of at least 5 cm of cervical dilatation at time of admission compare with 60.3% of women admitted in early labour in a sample of Australian women in a randomised controlled trial which also employed criterion of < 5 cm cervical dilatation [15].

The odds of oxytocic augmentation was more than three times higher for women admitted in early labour than those admitted in active labour, consistent with associations found in all [3, 4, 6, 9–12, 14] but one [2] other study comparing oxytocic augmentation rates. Our findings indicate an absolute 24–27% difference (depending on the cut-point for defining early labour) in rate of oxytocic augmentation between women admitted in early and active labour. Others have reported differences in the rate of augmentation between women admitted in early versus active labour from - 0.50% [2] to 47.0% [10, 14]. Our findings of the absolute differences in rates and the odds of oxytocic augmentation were persistent across the alternative definitions of early labour applied here.

Admission in early labour was associated with more than twice the odds of epidural use over and above the impact of parity and augmentation with oxytocin, which increased to almost four times the odds when applying new WHO criteria of < 5 cm cervical dilatation, with an absolute 25–28% difference in rate of epidural use between women admitted in early and active labour. Other research [1, 3, 6, 12] has consistently found that women admitted in early labour are more likely to have epidural analgesia, with differences in absolute rates of epidural analgesia use between women admitted in early and active labour from 2.4% [12] to 25.5% [1].

The association between timing of admission and length of maternal hospital stay was attenuated after accounting for mode of birth in this study, but retained significance even after adjustment when early labour was defined as < 5 cm cervical dilatation. Our concurrent finding that women admitted in early labour had 2.8–3.5 times the odds of caesarean than those admitted in active labour (after accounting for parity), and their infants had 1.5–1.6 times the odds of special care nursery admission, indicates a significant combined burden of early admission on both women and the healthcare system. Our finding that admission before 5 cm cervical dilatation was associated with 2.8 times the odds (95%CI 1.60, 5.05) of caesarean birth was consistent with findings from another study in Australia that employed the same criteria for early labour admission and found it to be associated with 2.4 times the odds of caesarean birth [15].

In general, extending the definition of early labour to include women with up to 5 cm cervical dilatation revealed associations between stage of labour at admission and a wider scope of outcomes than applying the previously recommended definition of up to 4 cm. Although associations were no longer apparent with caesarean for fetal distress, early labour at admission was significantly associated with artificial rupture of membranes, assisted vaginal birth, neonatal resuscitation, use of pharmacological pain relief and maternal length of stay after all additional adjustments, when these outcomes were not significantly associated with stage of labour at admission using a less inclusive definition of early labour.

Associations between early labour admission, increased obstetric intervention (and subsequent use of other hospital resources) and increased risk of adverse clinical outcomes has not been well understood [26]. It is possible that women who present and are admitted to hospital in early labour have an inherently higher risk of labour warranting medical intervention. It may also be that increased exposure to the medical system results in the amplification of risk for healthy labouring women [4].

Early labour admission is likely to be driven by interrelated factors. First, clinicians may have difficulty diagnosing active labour. There is little consensus in definitions of onset of any identified stages of labour in the research literature [17]. Lack of evidence-based approaches to clinical decision-making for diagnosing active labour has been identified in several countries [27]. Clinical judgements about the presence of active labour (and appropriateness of hospital admission) lack consistency between clinicians [28] and are usually made in busy clinical units with limited resources and emotional pressures that add complexity to the judgement [29]. Second, women may prefer hospital admission prior to active labour. Women experience uncertainty about recognising labour, determining progress, and hospital admission decision-making [29–32] and seek early hospital admission because of pain, anxiety and feeling unsupported during early labour [31, 33, 34]. Although maternity services routinely advise women to return home before active labour, women often prefer admission on initial presentation over repeated discharge [29]. In this study, we did not have the opportunity to assess women’s reasons for admission in early labour. Women may not be aware of the implications of early admission and/or lack confidence for managing pain or uncertainty at home.

A number of small trials have demonstrated that systematised clinician and consumer decision-making support tools can reduce rates of admission to hospital in early labour [16, 29, 35–39]. However, these studies are old, were conducted in non-representative samples and/or involve cost- and time-intensive interventions that are unsuitable for wide-scale implementation in a public hospital system. Currently in Australia, there are no standardised tools to guide clinicians’ or women’s decision-making about timing of admission. However, the combined evidence from this and other research provides sufficient basis for the development of decision tools that may improve the quality of decisions about timing of admission and prudent use of healthcare resources.

This is the first study to estimate the prevalence of early admission to hospital in labour in Australia. We employed rigorous quality assurance processes for the data extraction and found high consistency between data retrievers, and consulted with local clinicians to ensure the practical relevance of outcomes assessed. Nevertheless, there were some noteworthy limitations to our methods. First, we adopted a non-randomised, single-centre study design and limited our sample to women with live, singleton births equal to or greater than 37 weeks. While this was done purposefully to further control for anticipated confounding and optimise the comparability of our findings from Australia with previous research conducted internationally, we recommend caution in generalising our findings to all women. Second, we were limited by the data available in paper records in the study setting. While we assessed and statistically accounted for a several potential confounders of the association between early admission and outcomes, there may be others that remain unmeasured and unaccounted for (e.g., maternal anxiety). Third, it is possible that some women were categorised incorrectly for stage of labour at time of admission, given possible delays between timing of admission and first vaginal examination at which cervical dilation was recorded. If women presenting in early labour had moved to active labour at the time of first vaginal examination, they would have been misclassified as admitted in active labour, resulting in a bias towards no effect. We adjusted for parity in our analyses to reflect the odds of outcomes associated with stage of labour at hospital admission for all women, regardless of parity. Alternatively, we could have stratified our analyses consistent with clinical literature and conducted separate analyses by parity, but this would have resulted in complex probabilities to apply for specific subgroups that may threaten their usefulness for women’s decision making. Further formative work is needed to inform which scientific approaches provide findings that are most useful for all stakeholder groups, so that the type of evidence produced can be assured to meet the decision making needs of women in their timing of presentation to hospital in labour.

## Conclusions

This research provides preliminary evidence that more than half of women who had a live, singleton birth at equal to or greater than 37 weeks may be exposed to risks of avoidable medical intervention due to admission to hospital in early labour, potentially resulting in a substantial preventable burden on healthcare resources. These women and the clinicians caring for them may not be fully aware of these risks when making decisions about admission. Accordingly, antenatal information provision and advice about timing of presentation to hospital in labour should incorporate discussion of patient preferences regarding use or avoidance of these medical procedures and support for patients to execute an informed decision about early labour management. Research on reasons for early labour admission from the perspectives of all relevant parties (e.g., women, partners and support people, clinicians) and considering the role of hospital policies, clinical guidelines, and broader cultural factors may help to design effective support strategies for safe management of early labour outside the hospital setting and safe delays in hospital admission during labour.

We are not aware of any established sets of performance measures or core process or outcome minimum reporting standards that incorporate timing of admission to hospital in labour. Given the burden of avoidable healthcare resource use associated with early labour admission and its negative impact on women’s and babies’ outcomes and experiences, routine and more widespread reporting of timing of admission in labour is warranted.

## Data Availability

The datasets used and/or analysed during the current study are available from the corresponding author on reasonable request.

## Abbreviations

AOR: Adjusted Odds Ratio
CI: Confidence Interval
cm: centimetres
mL: millilitre
WHO: World Health Organisation

## Footnotes

1 July 2013 to June 2014, March 2015 to June 2015, and October 2015 to December 2015. The data collection periods were selected to allow secondary usage of the data for a different project utilising a time series design for evaluating upcoming service changes.

## References

[1] Lundgren I, Andrén K, Nissen E, Berg M (2013). Care seeking during the latent phase of labour - frequencies and birth outcomes in two delivery wards in Sweden. Sex Reprod Healthc.

[2] Rahnama P, Ziaei S, Faghihzadeh S (2006). Impact of early admission in labor on method of delivery. Int J Gynecol Obstet.

[3] Holmes P, Oppenheimer LW, Wu WS (2001). The relationship between cervical dilatation at initial presentation in labour and subsequent intervention. Br J Obstet Gynaecol.

[4] Bailit JL, Dierker L, Blanchard MH, Mercer BM (2005). Outcomes of women presenting in active versus latent phase of spontaneous labor. Obstet Gynecol.

[5] Janna JR, Chowdhury SB (2013). Impact of timing of admission in labour on subsequent outcome. Commun Based Med J.

[6] Rota A, Antolini L, Colciago E, Nespoli A, Borrelli SE, Fumagalli S (2018). Timing of hospital admission in labour : latent versus active phase , mode of birth and intrapartum interventions . A correlational study. Women and Birth.

[7] National Institute for Health and Clinical Excellence (2016). Intrapartum care for healthy women and babies (CG190).

[8] NSW Health (2010). Maternity - towards Normal birth in NSW. Sydney.

[9] Hemminki E, Simukka R (1986). The timing of hospital admission and progress of labour. Eur J Obstet Gynecol Reprod Biol.

[10] Mikolajczyk R, Zhang J, Chan L, Grewal J (2008). Early versus late admission to Labor/Delivery, labor progress and risk of caesarean section in nulliparous women. Am J Obstet Gynecol.

[11] Albassam AN (2010). The outcome of latent phase vs. active phase admission to labour room of low risk nulliparous women in labour. J Fac Med Baghdad.

[12] Neal JL, Lamp JM, Buck JS, Lowe NK, Gillespie SL, Ryan SL (2014). Outcomes of nulliparous women with spontaneous labor onset admitted to hospitals in preactive versus active labor. J Midwifery Women Heal.

[13] Jackson DJ, Lang JM, Ecker J, Swartz WH, Heeren T (2003). Impact of collaborative management and early admission in labor on method of delivery. J Obstet Gynecol Neonatal Nurs.

[14] Mikolajczyk RT, Zhang J, Grewal J, Chan LC, Petersen A, Gross MM (2016). Early versus Late Admission to Labor Affects Labor Progression and Risk of Cesarean Section in Nulliparous Women. Front Med.

[15] Davey MA, McLachlan HL, Forster D, Flood M (2013). Influence of timing of admission in labour and management of labour on method of birth: results from a randomised controlled trial of caseload midwifery (COSMOS trial). Midwifery.

[16] Mcniven PS, Williams JI, Hodnett E, Kaufman K, Hannah ME (1998). An early labor assessment program : a randomized , Controlled Trial. Birth.

[17] Hanley GE, Munro S, Greyson D, Gross MM, Hundley V, Spiby H (2016). Diagnosing onset of labor: a systematic review of definitions in the research literature. BMC Pregnancy Childbirth.

[18] WHO (2003). WHO Surgical Care at the District Hospital.

[19] World Health Organization (2018). Intrapartum care for a positive childbirth experience.

[20] Tracy S, Tracy M (2003). Costing the cascade: estimating the costs of increased intervention in childbirth using population data. Br J Obstet Gynaecol.

[21] Queensland Health (2015). Patient safety unit [internet].

[22] Lowe NK (2007). A review of factors associated with dystocia and cesarean section in nulliparous women. J Midwifery Women Heal.

[23] Liddy C, Wiens M, Hogg W (2011). Methods to achieve high Interrater reliability in data collection from. Ann Fam Med.

[24] Shrout PE, Fleiss JL (1979). Intraclass Correlations : Uses in Assessing Rater Reliability. Psychol Bul.

[25] Shrout PE, York N (1998). Measurement reliability and agreement in psychiatry. Stat Methods Med Res.

[26] Marowitz A (2014). Caring for women in early labor: can we delay admission and meet women’s needs?. J Midwifery Women Heal.

[27] Lauzon L, Hodnett ED. Labour assessment programs to delay admission to labour wards. Cochrane Database Syst Rev. 2001(3):CD000936. 10.1002/14651858.CD000936. 10.1002/14651858.CD000936 11686969

[28] Gross MM, Burian RA, Frömke C, Hecker H, Schippert C, Hillemanns P (2009). Onset of labour: Women’s experiences and midwives’ assessments in relation to first stage duration. Arch Gynecol Obstet.

[29] Cheyne H, Hundley V, Dowding D, Bland JM, McNamee P, Greer I (2008). Effects of algorithm for diagnosis of active labour: cluster randomised trial. BMJ.

[30] Cheyne H, Terry R, Niven C, Dowding D, Hundley V, Mcnamee P (2007). ‘Should I come in now?’: A study of women’s early labour experiences. Br J Midwifery.

[31] Carlsson I-M, Hallberg LR-M, Petterson K (2009). Swedish women’s experiences of seeking care and being admitted during the latent phase of labour : a grounded theory study. Midwifery.

[32] Beebe KR, Humphreys J (2006). Expectations, perceptions, and Management of Labor in Nulliparas prior to hospitalization. J Midwifery Women Heal..

[33] Eri TS, Blystad A, Gjengedal E, Blaaka G (2010). Negotiating credibility: first-time mothers’ experiences of contact with the labour ward before hospitalisation. Midwifery.

[34] Barnett C, Hundley V, Cheyne H, Kane F (2008). ‘Not in labour’: impact of sending women home in the latent phase. Br J Midwifery.

[35] Hodnett ED, Stremler R, Willan AR, Weston JA, Lowe NK, Simpson KR (2008). Effect on birth outcomes of a formalised approach to care in hospital labour assessment units: international, randomised controlled trial. BMJ.

[36] Janssen PA, Iker CE, Carty EA. Early labour assessment and support at home: a randomized controlled trial. J Obstet Gynaecol Can. 2003;25(9):734–41 Available from: https://www.embase.com/search/results?subaction=viewrecord&from=export&id=L137572425%5Cn. 10.1016/s1701-2163(16)31002-7 12970808

[37] Janssen PA, Still DK, Klein MC, Singer J, Carty EA, Liston RM (2006). Early labor assessment and support at home versus telephone triage: a randomized controlled trial. Obstet Gynecol.

[38] Lauzon L, Hodnett ED. Antenatal education for self-diagnosis of the onset of active labour at term. Cochrane Database Syst Rev. 1998;(4):CD000935. 10.1002/14651858.CD000935. 10.1002/14651858.CD000935 PMC6483815 10796218

[39] Bonovich L (1990). Recognizing the onset of labor. J Obstet Gynecol Neonatal Nurs.

